# Upregulation of TRPC5 in hippocampal excitatory synapses improves memory impairment associated with neuroinflammation in microglia knockout IL-10 mice

**DOI:** 10.1186/s12974-021-02321-w

**Published:** 2021-11-26

**Authors:** Shiji Huo, Jiling Ren, Yunqing Ma, Ahsawle Ozathaley, Wenjian Yuan, Hong Ni, Dong Li, Zhaowei Liu

**Affiliations:** 1 Medical School, Nankai University, No.94, Weijin Road, Nankai District, Tianjin, 300071 China; 2 Tianjin Key Laboratory of Tumor Microenvironment and Neurovascular Regulation, Nankai University, Tianjin, China; 3 Department of Pathogen Biology, Basic Medical School, Tianjin Medical University, Tianjin, China

**Keywords:** IL-10, Microglia, TRPC5, NLRP3, Memory

## Abstract

**Background:**

Members of the transient receptor potential canonical (TRPC) protein family are widely distributed in the hippocampus of mammals and exert respective and cooperative influences on the functions of neurons. The relationship between specific TRPC subtypes and neuroinflammation is receiving increasing attention.

**Methods:**

Using Cx3cr1^CreER^IL-10^−/−^ transgenic mice and their littermates to study the relationship between TRPC channels and memory impairment.

**Results:**

We demonstrated that Cx3cr1^CreER^IL-10^−/−^ mice displayed spatial memory deficits in object location recognition (OLR) and Morris water maze (MWM) tasks. The decreased levels of TRPC4 and TRPC5 in the hippocampal regions were verified via reverse transcription polymerase chain reaction, western blotting, and immunofluorescence tests. The expression of postsynaptic density protein 95 (PSD95) and synaptophysin in the hippocampus decreased with an imbalance in the local inflammatory environment in the hippocampus. The number of cells positive for ionized calcium-binding adaptor molecule 1 (Iba1), a glial fibrillary acidic protein (GFAP), increased with the high expression of interleukin 6 (IL-6) in Cx3cr1^CreER^IL-10^−/−^ mice. The nod-like receptor protein 3 (NLRP3) inflammasome was also involved in this process, and the cytokines IL-1β and IL-18 activated by NLRP3 were also elevated by western blotting. The co-localization of TRPC5 and calmodulin-dependent protein kinase IIα (CaMKIIα) significantly decreased TRPC5 expression in excitatory neurons. AAV9-CaMKIIα-TRPC5 was used to upregulate TRPC5 in excitatory neurons in the hippocampus.

**Conclusions:**

The results showed that the upregulation of TRPC5 improved the memory performance of Cx3cr1^CreER^IL-10^−/−^ mice related to inhibiting NLRP3 inflammasome-associated neuroinflammation.

**Graphical Abstract:**

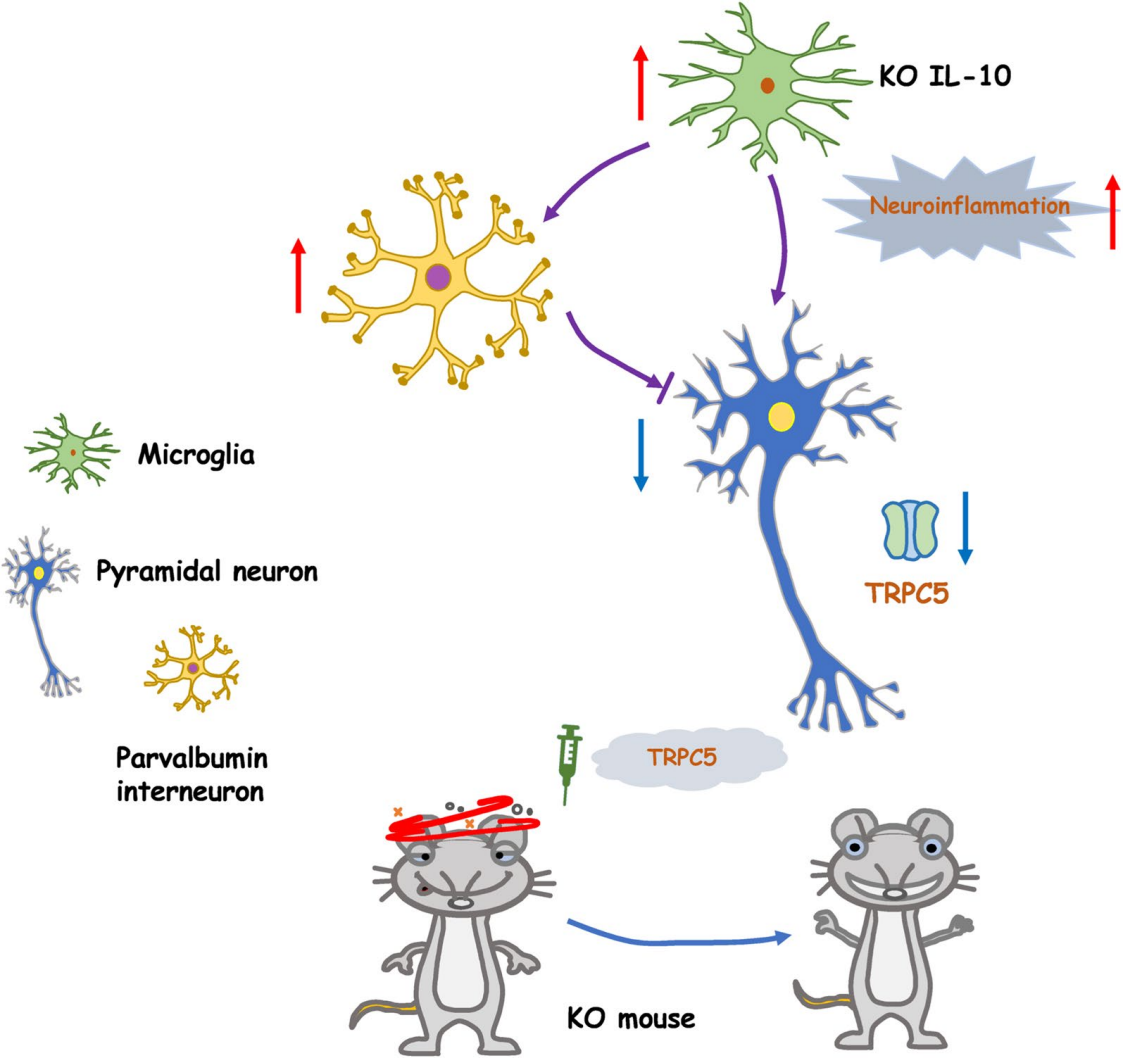

**Supplementary Information:**

The online version contains supplementary material available at 10.1186/s12974-021-02321-w.

## Introduction

The TRPC protein family has seven members (TRPC1 to TRPC7) that form homo- and/or heteromorphic tetramers. TRPC is a non-selective cationic membrane channel with Ca^2+^ permeability. According to sequence homology, the family can be divided into three subfamilies: TRPC1, C4, and C5; TRPC3, C6, and C7; and TRPC2, which is a pseudogene in humans [1].

The physiological and pathological functions of TRPC, particularly in the central nervous system (CNS), are of increasing concern due to the extensive localization of TRPC, especially in the hippocampus. Evidence is converging to show the involvement of TRPC channels in cognitive functions, since multiple TRPC subtypes are highly expressed in the hippocampus.

TRPC1 is indispensable for environmental enrichment-induced spatial memory enhancement, which is related to long-term potentiation (LTP) induction and hippocampal neurogenesis [2]. The function of the TRPC1/4/5 subfamily plays a key role in spatial working memory formation [3], since TRPC1/4/5^−/−^ mice exhibited deficiencies in adapting to a new challenge in a relearning task.

Neuroinflammation is a complex response to brain injury and a major contributor to progressive neuronal damage. IL-10 is a major anti-inflammatory cytokine that maintains the balance of the immune response and is an important molecule in the modulation of neuronal homeostasis and cell survival. At the level of the hippocampus, it has been shown that IL-10 plays a key role in improving the learning and memory ability of animals under physiological and pathological conditions [4]. IL-10 helped to improve spatial memory performance in Sprague–Dawley rats treated with *Escherichia coli* [5]. Increased IL-10 levels played a role in the process of enriched environment alleviated LPS-induced spatial learning and memory impairment [6]. IL-10 is an important molecule in the modulation of learning and memory dysfunction, as shown by the IL-10^tm1/tm1^ mice that exhibited behavioral deficits in the MWM test [7].

Microglia are the main innate immune cells in the CNS and play an important role in neuroinflammation [8]. Microglia in the brain and are the main cytokine producers, including IL-10. Many studies have confirmed that IL-10 secreted by microglial cells plays a key role in the pathological process of neuroinflammation [9, 10]. Neuroinflammation can trigger cognitive impairment, and IL-10 can alleviate the harmful effects of neuroinflammation on memory and plasticity [11–13]. The role of inflammasome nod-like receptor protein 3 (NLRP3) processes involved in recognition impairment in a variety of nervous system diseases, has been supported by experimental evidence, especially in recent years. Several studies have shown that NLRP3 can be a target for improving memory impairment in diabetes [14–16], sepsis-associated encephalopathy [17], hypoxemia [18], epilepsy [19, 20], Alzheimer’s disease (AD) [21, 22], intracerebral hemorrhage [23], cerebral ischemia [24], and aged [25].

In our study, we focused on the effects of IL-10 induced from microglial cells on animal cognitive and behavioral abilities to explore the role of TRPC and NLRP3 in the hippocampus during this process.

## Materials and methods

### Animals

**Fig. 1:**
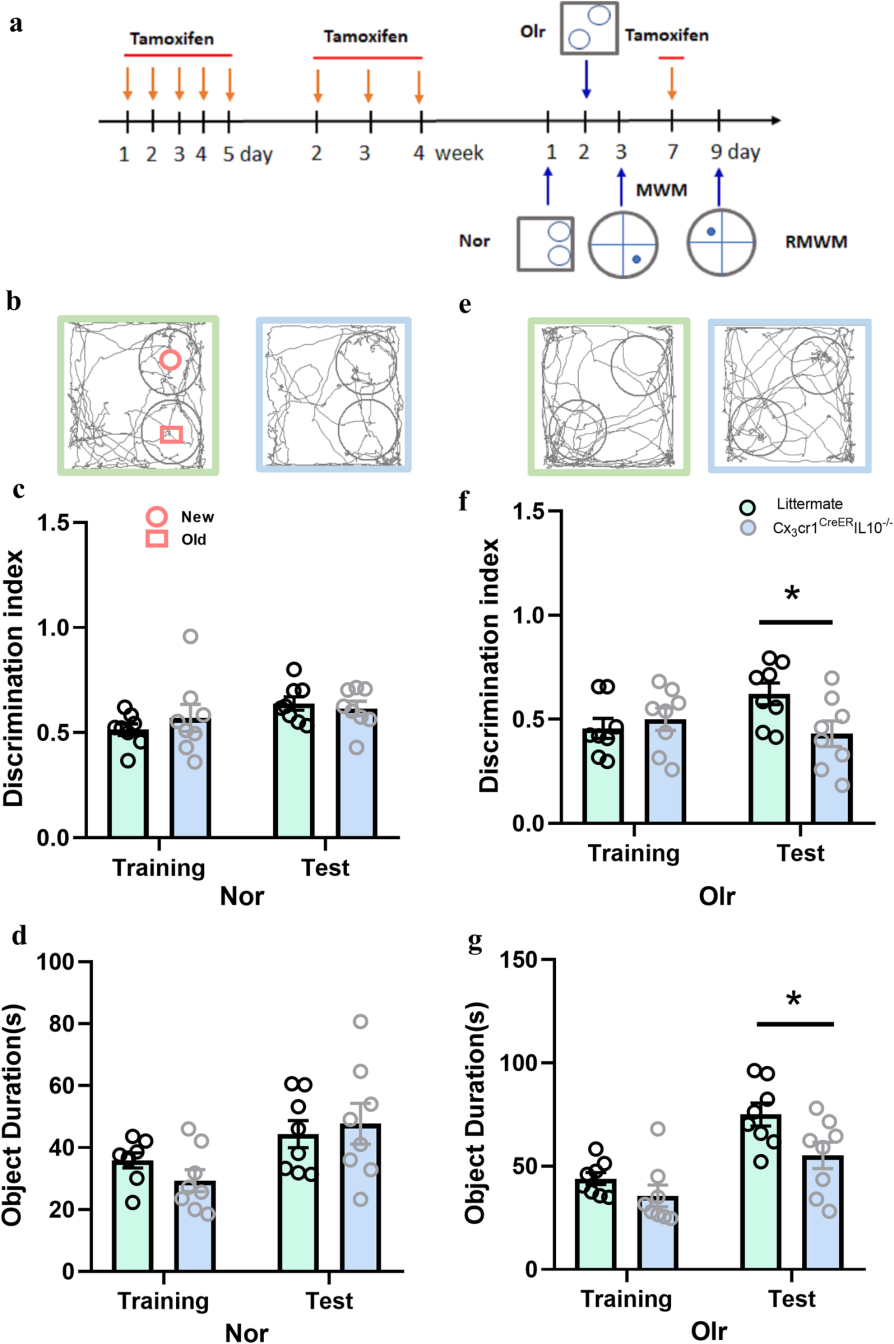
Cx3cr1^CreER^IL-10^−/−^mice showed a decrease recognition impairment in OLR task. **a** Schematic of experimental design and schedule. The animal experimental protocol indicated the time course of various interventions utilized during the experiment. **b** Representative movement traces from the two groups on the test stage of the NOR task. There were no significant differences between the two mice in the different groups. There was no significant difference between the two groups in the discrimination index (**c**) and the new object duration (**d**). **e** Representative movement traces from the two groups on the test stage of the OLR task. There was a significant decrease in the location of new objects in the Cx3cr1^CreER^IL-10^−/−^ group. There was a significant decrease in the two groups in discrimination index (**f**) and new object duration (**g**) in the test stage in the Cx3cr1^CreER^IL-10^−/−^ group. Each dot represents a mouse. Bars represent mean ± SEM. *n* = 8 in each group. Significant differences were established by *t*-test, *P < 0.05

All experiments were performed on male mice that were 8–10 weeks old and weighed 22–26 g. All animals were on a C57BL/6 background and were maintained in a reversed 12-h light–dark cycle with free access to food and water. Tamoxifen-inducible Cx3cr1^CreER^ with IL-10^flox/flox^ mice were bred, hereafter denoted as Cx3cr1^CreER^IL-10^−/−^. Both littermate and Cx3cr1^CreER^IL-10^−/−^ mice was administered with tamoxifen (Sigma-Aldrich, MO, USA) as a solution in corn oil (20 mg/ml) by intraperitoneal injection (80 μl per mouse) [26, 27]. Treatment with tamoxifen for five consecutive days eliminated IL-10 production by microglia, after that receiving an additional tamoxifen dose once a week to deplete IL-10 in newly made microglia [26] (Fig. 1a). All procedures and protocols for this study were approved by the Ethical Commission of Nankai University based on the NIH Guide for the Care and Use of Laboratory Animals. B6.129-Il10tm1.1 (Flox) Smoc mice and Cx3cr1^CreER^ transgenic mice were purchased from Shanghai Model Organisms Center, Inc.

### Novel object recognition (NOR) task and OLR task

Each mouse was gently handled for 3 min every day for three consecutive days before the behavioral test. The test was conducted in a bare square box (48 cm long, 48 cm wide, 36 cm high) made of compressed wood. Briefly, the NOR and OLR tasks consisted of two sessions: the training phase and the test phase. In the NOR task, during the test period, the mice were placed in the empty box and allowed to explore freely for 5 min to adapt to the environment. Two identical objects (plastic boxes, 4–5 cm high) were arranged in a straight line along one side of the wall, 8 cm from the sides. The experimental mice were placed in the box facing the opposite wall and were free to explore and adapt for 5 min. After a 2-h interval, the animals were reintroduced into the experimental box for free exploration during the experimental period. At this point, one of the two objects used during training was replaced by an object of similar size. If any of the mice pointed or touched the new object with its nose within 1 cm, it was considered exploratory. The objects were thoroughly cleaned between trials to avoid olfactory cues. The mice were tracked using a charge-coupled device camera connected to a personal computer (Ethovision 2.0, Noldus, Wageningen, Netherlands).

In the OLR task, the experimental procedure was similar to the NOR task in the training stage. The difference was that in the test stage, the two unchanged objects were placed diagonally, as shown in Fig. 1.

During the training phase in both the NOR and OLR tasks, if the mouse total exploration time for the two objects was less than 10 s in 5 min, the data were eliminated in a later study. The discrimination index (DI) and object duration were used as the NOR and OLR task evaluation index. The DI is the percentage of time each mouse spent exploring new objects and positions.

### MWM task and RMWM task

The mice swam freely in the pool without platform 90 s on 1 day before the test, so that they could got familiar with the maze environment. In the training stage, each mouse was placed into the water with their heads facing the wall, and randomly selected one of the four starting positions of east, west, south and north. Record the time it takes the animal to find the underwater platform (escape latency). If the escape latency exceeded 60 s, the mouse was guided to the platform and stayed on the platform for 10 s.

Training was conducted once a day at each of the four entry points, and the results of the day were statistically analyzed using the mean value of the four escape latencies. The training lasted for four days.

**Fig. 2:**
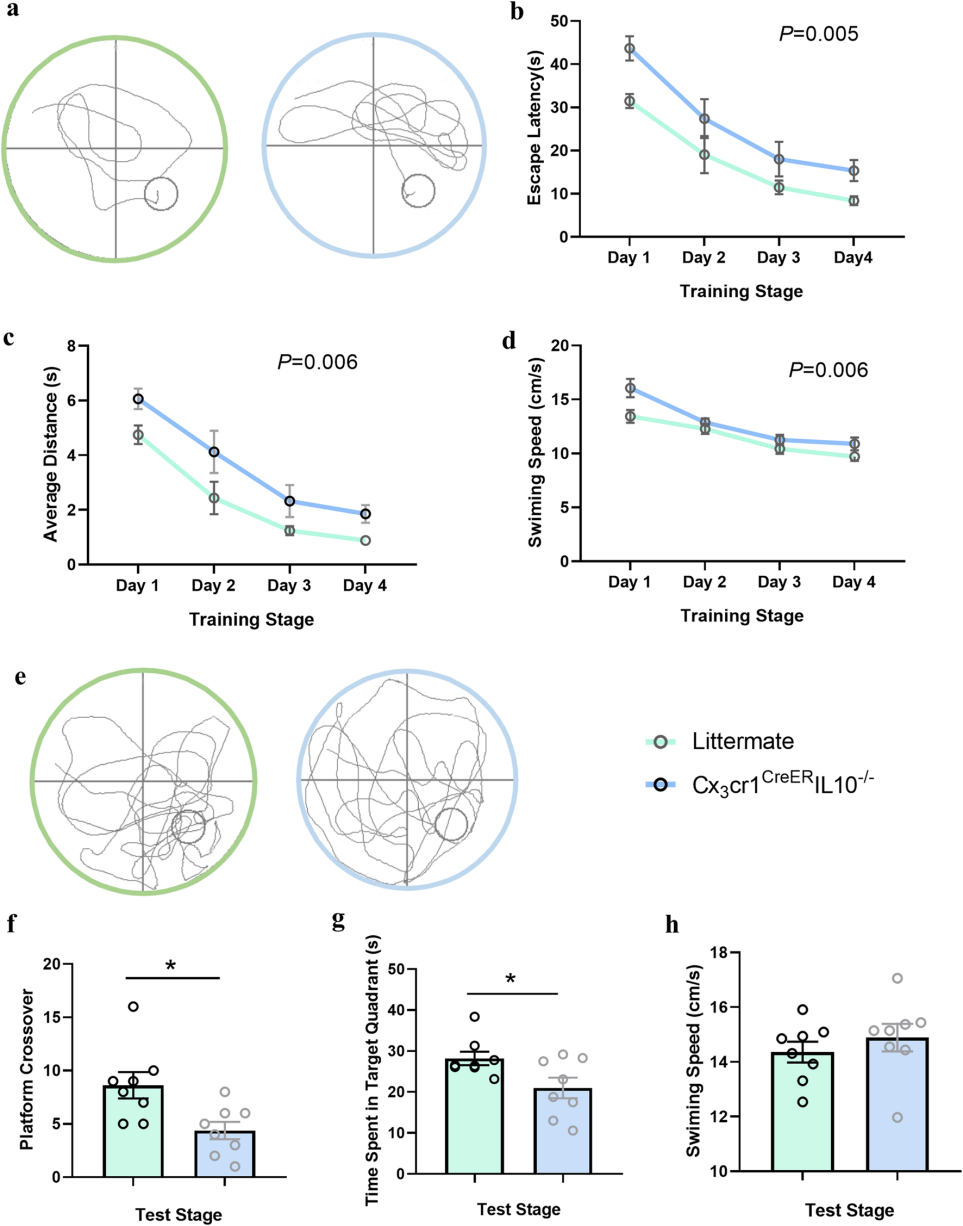
Cx3cr1^CreER^ IL-10^−/−^mice showed a decrease recognition impairment in MWM task. **a** Representative movement traces from two groups during the training stage of the MWM task. Cx3cr1^CreER^IL-10^−/−^mice had more dispersed paths in the training stage, suggesting impairments in learning ability. There was a significant increase in escape latency (**b**) and average distance (**c**) in Cx3cr1^CreER^IL-10^−/−^ mice in the training stage, while Cx3cr1^CreER^IL-10^−/−^ mice swam faster in the MWM task (**d**). **e** Representative movement traces from the two groups on the test stage of the MWM task. Cx3cr1^CreER^IL-10^−/−^mice had more dispersed paths in the test stage, suggesting memory impairments. There was a significant decrease in both the platform crossover times (**f**) and time spent in the target quadrant (**g**) in the Cx3cr1^CreER^IL-10^−/−^ group in the test stage, while the swimming speed was similar between the two groups during the test stage (**h**). Each dot represents a mouse. Bars represent mean ± SEM. *n* = 8 in each group. Significant differences were established by two-way ANOVA (**b**–**d**) and *t*-test in other bar graphs, *P < 0.05

The concealed platform was removed 24 h after the test. After the mice were placed in the water, their swimming trails for 60 s were recorded, and the residence time of the mice in the original platform quadrant and the times of platform crossover were statistically analyzed. The reversal phase started after the test, and the platform was moved to the opposite quadrant of the tank. The platform remained in this northwest quadrant location for all training trials on days 1, 2, and 3, but not for day 4 of the test trial (Fig. 2 and Additional file 1: Fig. S2). The swimming activity of each mouse was automatically recorded using a video tracking system (Ethovision 2.0, Noldus, Wageningen, Netherlands).

### Quantitative PCR

Total RNA was extracted from hippocampal tissues with Trizol (Biosharp, China). The Total RNA was reverse transcribed into complementary DNA (cDNA) by using the SuperScript First-Strand Synthesis System for RT-PCR (Invitrogen). The primers used to measure gene expression are the following TRPC1 Forward-CGTGCGACAAGGGTGACTATTAT, Reverse-TGCATCTGCGGACTGACAAC; TRPC3 Forward-ACCCTGCTTTTACCACGGTT, Reverse-GCATGTTGAGCAGAACGACC; TRPC4 Forward-AAACCCCATCGGAACTGACC, Reverse-GCTAGTCCATCATCTCCGCA; TRPC5 Forward-TTTGCCAACGGACTGAACCA, Reverse-GAAGGGTTTCAAAGAGCGTGG; TRPC6 Forward-AAGTGAACGAAGGGGAGCTG, Reverse-ACAGTCTCTCCCCAAGCTTTC; TRPC7 Forward-TCCCTTTAACCTGGTGCCGAGTC, Reverse-TTCAGCATGCCCATTTCCAGG; β-actin Forward-CGGTTCCGATGCCCTGAGGCTCTT, Reverse-CGTCACACTTCATGATGGAATTGA.

### Golgi staining

Samples were treated according to instructions of the Rapid Golgistain^TM^ Kit (FD, Inc., USA). Coronal sections (100 μm in thickness) including hippocampal structures were prepared using a freezing microtome (Leica CM 1860, Germany). The Golgi staining images were taken using a microscope (Olympus, BX53, Japan) at a magnification of 100 × oil immersion. Average spine densities were calculated from at least 6 separate image stacks per animal. Image J software (NIH, MD, USA) was used for the morphometric analysis of digitized images.

### Western blotting

Protein sample concentrations were measured using the Pierce BCA Protein Assay Kit (Biosharp, Hefei, China). Equal amounts of proteins (10 μg per lane) were run on 10 or 12% SDS-PAGE and then transferred to PVDF membranes by electroblotting (Bio-Rad, Hercules, CA, USA). The PVDF membranes were blocked with 5% skim milk powder (Becton Dickinson, CA, USA) diluted with Tween/0.1 M PBS (TBST) for one h at room temperature before incubation with the primary antibody.

The membranes were washed with TBST three times for 10 min after the primary antibodies were incubated at 4 °C overnight. The membranes were then incubated with secondary antibodies for 1 h at room temperature, washed three times for 10 min in TPBS, and reacted with chemiluminescent substrate (Biosharp, Hefei, China). The bands were obtained using an ECL luminescence imaging system (Tanon 5200, China). The densities of the target protein bands were measured using Image J and normalized to corresponding β-actin bands. All the antibodies used are listed in Table 1.

**Table 1:** Antibodies

Antibody	Species/clonality	Source (Catalogue No.)	Dilution	Usage
Anti-NMDAR2A	Rabbit/polyclonal	Abcam/ab124913	1:1000/1:200	WB/IF
Anit-NMDAR2B	Rabbit/polyclonal	Abcam/ab65783	1:1000/1:200	WB/IF
Anti-GAD67	Mouse/monoclonal	Abcam/ab26116	1:1000	WB
Anti-TRPC1	Rabbit/polyclonal	Proteintech/19482-1-AP	1:1000/1:200	WB/IF
Anti-TRPC3	Rabbit/polyclonal	CST/77934	1:1000/1:200	WB/IF
Anti-TRPC4	Rabbit/polyclonal	Abcam/ab83689	1:1000/1:200	WB/IF
Anti-TRPC5	Rabbit/polyclonal	Proteintech/25890-1-AP	1:1000/1:200	WB/IF
Anti-TRPC6	Rabbit/polyclonal	Abcam/ab101622	1:1000/1:200	WB/IF
Anti-SYP	Rabbit/polyclonal	Beyotime/AF8091	1:1000/1:200	WB/IF
Anti-PSD95	Rabbit/polyclonal	Abcam/ab18258	1:1000/1:200	WB/IF
Anti-parvalbumin	Rabbit/polyclonal	Abcam/ab11427	1:1000	WB
Anti-β-Actin	Rabbit/polyclonal	Abcam/ab8227	1:3000	WB
Anti-Camkllα	Mouse/monoclonal	Abcam/ab22609	1:200	IF
Anti-TNF-α	Rabbit/polyclonal	Abcam/ab9739	0.1 μg/ml	WB
Anti-Iba-1	Rabbit/polyclonal	Fujifilm/PTK1381	1:1000/1:200	WB/IF
Anti-Iba-1	Mouse/monoclonal	Proteintech/66827-1-Ig	1::200	IF
Anti-IL-1β	Rabbit/polyclonal	Abcam/ab9722	1:100	WB
Anti-IL-6	Rabbit/polyclonal	Abcam/ab208113	1:1000	WB
Anti-IL-10	Mouse/monoclonal	Proteintech/60269	1:200	IF
Anti-IL-10	Rabbit/polyclonal	Abcam/ab9969	0.1 μg/ml	WB
Anti-GFAP	Rabbit/polyclonal	Ab33922/ab7260	1:1000/1:200	WB/IF
Anti-Nlrp3	Rabbit/polyclonal	Wanleibio/WLH3383	1:1000	WB
Anti-IL-18	Rabbit/polyclonal	Wanleibio/WL01127	1:1000	WB
Anti-IL-18	Rabbit/polyclonal	Proteintech/10663-1-AP	1:1000	WB
Anti-caspase-1	Rabbit/monoclonal	Beyotime/AF1681	1:1000	WB
Anti-ASC	Rabbit/monoclonal	Proteintech/67494-1-Ig	1:1000	WB
HRP-conjugated	Anti-mouse IgG	Abcam/ab205719	1:5000	WB
HRP-conjugated	Anti-rabbit IgG	Abcam/ab205718	1:5000	WB
Alexa Fluor 488-conjugated anti-mouse Ig G	Goat	Proteintech/SA00013-2	1:200	IF
Alexa Fluor 594-conjugated anti-rabbit IgG	Goat	Proteintech/SA00013-4	1:200	IF
Alexa Fluor 594-conjugated anti-mouse IgG	Goat	Proteintech/SA00013-3	1:200	IF

### Immunostaining

Cx3cr1^CreER^IL-10^−/−^ (*n* = 3) and littermate (*n* = 3) male mice were killed and perfused with PBS (pH = 7.4), followed by 4% paraformaldehyde in PBS. The samples were immersed in a fixed solution overnight and then dehydrated in a gradient solution of 10, 20, and 30% sucrose in PBS. Each brain was embedded in O.C.T and coronal sections of 20 μm thickness were prepared using a freezing microtome (Leica CM 1860, Germany). The slices were washed three times with PBS for 5 min before staining. The tissue sections were permeabilized in 0.3% Triton X 100 for 30 min and then blocked in 10% normal goat serum. The blocked slices were incubated with the corresponding primary antibodies overnight at 4 °C. The samples were washed in PBS three times for 5 min, and then incubated with fluorescent secondary antibodies for 1 h at room temperature. After being washed in PBS three times for 5 min, the slices were incubated with DAPI (Beyotime, China, 1:5000). Representative images were obtained using a fluorescence microscope (Olympus, BX53, Japan) or confocal microscopy (Olympus, FV1000, Japan). All the antibodies used in immunostaining test are shown in Table 1.

### ELISA analysis of IL-1β and IL-18

The hippocampus was collected and rinsed with PBS to remove excess blood, cut into 1–2 mm pieces and homogenized with a tissue homogenizer.  A total of 1.0 mL of cracking buffer (R&D Systems) was added. The brains were cleaved at room temperature under mild agitation for 30 min and centrifuged to remove fragments. The levels of IL-1β and IL 18 were determined by specific ELISA kits according to manufacturer instructions (R&D Systems, Inc., Minneapolis, MN, USA).

### AAV injection

The hippocampal region was injected with AAV vectors that included AAV9-CaMKIIα promoter-TRPC5 2928 (9.7 × 10^13^ μg/ml) or AAV9-GFP. A total of 10 mice received AAV9-TRPC5, and another 10 mice received AAV9-GFP. All vectors were generated and titered using Vigene Biosciences (Vigene Biosciences, Inc., China).

After the mice were anesthetized (10% chloral hydrate, 3.5 mL/kg), their brains were fixed to a stereotactic locator and the virus was injected into the bilateral middle regions of the hippocampus (0.5 μl/ hemisphere) using a 2 μl microsyringe according to the following stereotaxic coordinates referenced in mm from the bregma (AP = − 2 mm; ML =  ± 1.4 mm; DV = − 1.5 mm). Following injection, the microsyringe was left in place for 5 min to prevent backflow of the solution. After surgery, the mice were single-housed for one week to recover well.

### Data analysis

All data were expressed as mean ± SEM. One-way or two-way ANOVA were used for data analysis based on different experimental designs or datasets followed by multiple comparison test or by unpaired, two-tailed *t*-test. Values of *P* < 0.05 were considered statistically significant.

## Results

### Microglia knockout (KO) IL-10 impaired the learning and memory ability of mice

Before the experiment, all mice were detected by PCR and classified (Additional file 1: Fig. S1a). To test the learning and memory abilities of mice, we tested NOR and OLR in our study. Figure 1a shows the experimental procedure. There was no difference between the two groups during NOR detection in both the training and test stages, not only in the discrimination index, but also in object duration time history detection (*P* > 0.05, *n* = 8, *t*-test, Fig. 1b–d).

During the OLR task, Cx3cr1^CreER^IL-10^−/−^ mice showed reduced memory capacity. In the test stage, discrimination index (*t* = 2.387, *P* = 0.031 < 0.05, *n* = 8, *t*-test,) and object duration (*t* = 2.357, *P* = 0.034 < 0.05, *n* = 8, *t*-test) decreased in the Cx3cr1^CreER^IL-10^−/−^ group (Fig. 1e–g).

There was no difference between the two groups in NOR, but there was a difference in OLR, indicating that the two groups had differential sensitivity in spatial location recognition [28]. We further detected the learning and memory abilities of the two groups using MWM. The results showed that both the escape latency to the platform [*F* (1, 14) = 10.96, *P* < 0.01, two-way ANOVA, Fig. 2b] and the average distance [*F* (1, 14) = 10.36, *P* < 0.01, two-way ANOVA, Fig. 2c] in the target quadrant increased in KO group during the learning stage. In the test stage, the mice in the KO group showed a decrease in both platform crossover times (*P* < 0.05, *n* = 8, *t*-test) and time spent in the target quadrant (*P* < 0.05, *n* = 8, *t*-test, Fig. 2f). In the reversal MWM test, the KO mice showed a decreased ability in the learning stage, but not in the memory stage (Additional file 1: Fig. S2).

### TRPC4 and TRPC5 decreased in hippocampi of Cx3cr1^CreER^IL-10^−/−^ mice

**Fig. 3:**
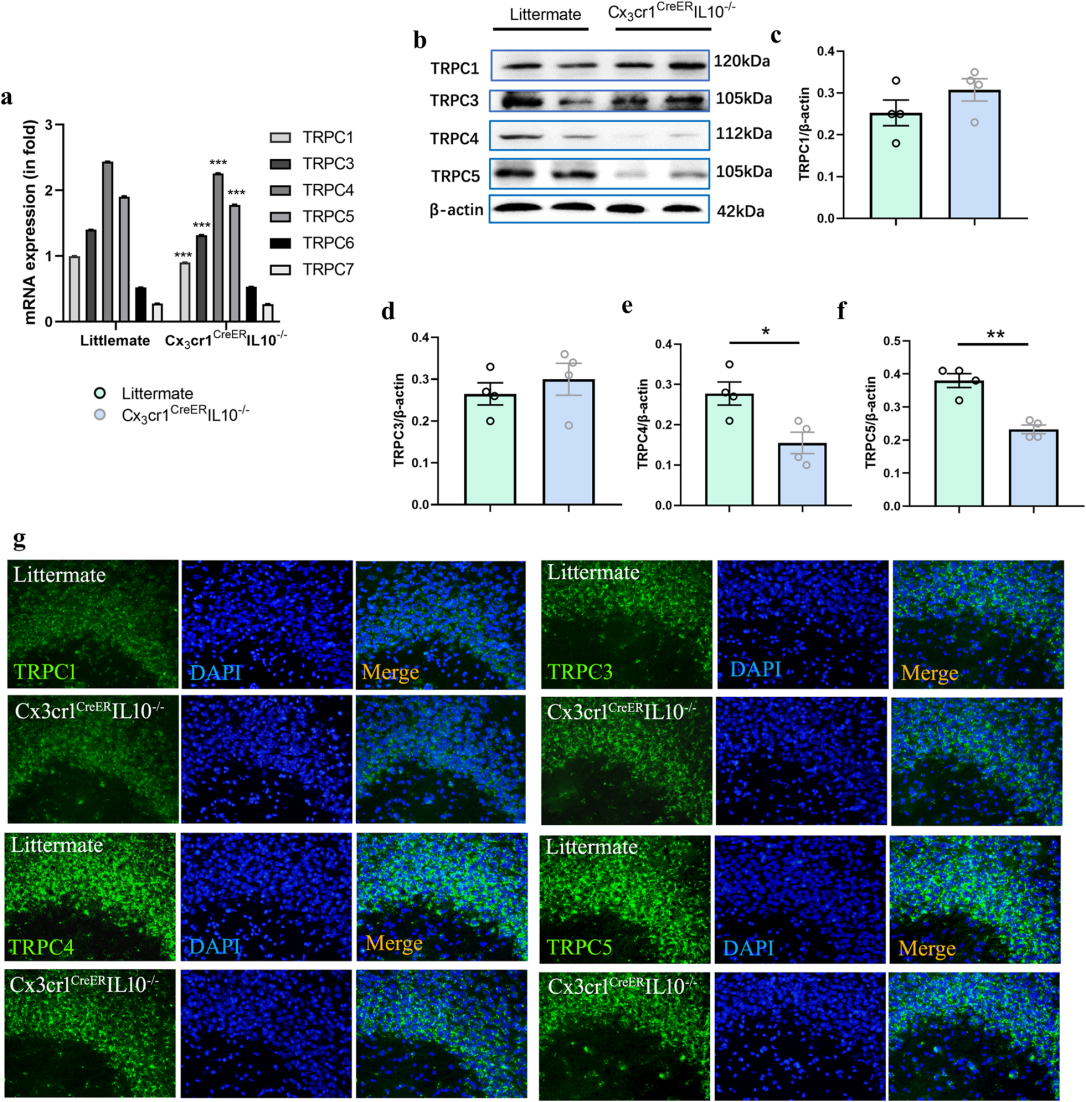
The decrease of both TRPC4 and TRPC5 in the hippocampi of Cx3cr1^CreER^ IL-10^−/−^mice. **a** RT-PCR results of **T**RPC isoforms in the hippocampus (*n* = 3 in each group, *t*-test). The marked significance is the comparison of the same isoform in the Cx3cr1^CreER^ IL-10^−/−^ with the littermate. **b** western blotting bands of TRPC1, TRPC3, TRPC4, and TRPC5 in littermates and in Cx3cr1^CreER^ IL-10^−/−^mice. Bar graphs represent densitometric plots of protein expression in littermate and Cx3cr1^CreER^ IL-10^−/−^mice in TRPC1 (**c**), TRPC3 (**d**), TRPC4 (**e**), and TRPC5 (**f**). Each dot represents a mouse. Bars represent mean ± SEM. *n* = 4 in each group, Significant differences were established by *t*-test. **g** Expression of TRPC1, TRPC3, TRPC4, and TRPC5 in the CA3 region of mouse hippocampal slices. Immunofluorescence images were captured with a 20 × objective. Green, immunoreactivity of TRPC1, TRPC3, TRPC4, and TRPC5; blue, nuclei stained with DAPI. Merged images of each TRPC isoform and DAPI staining. *P < 0.05, **P < 0.01, ***P < 0.001

Since spatial learning relies heavily on hippocampal activity, in addition to TRPC2, other TRPC subtypes were detected via RT-PCR since TRPC2 is not expressed in the hippocampus [29]. RT-PCR results showed that the mRNA levels of TRPC1, 3, 4 and 5 decreased in the hippocampus (Fig. 3a). Using western blot to detect the protein expression levels of these TRPC subtypes, also revealed that TRPC4 (*t* = 3.130, *P* = 0.020 < 0.05, *n* = 4, *t*-test) and TRPC5 (*t* = 5.910, *P* = 0.001 < 0.01, *n* = 4, *t*-test) were decreased in the hippocampus (Fig. 3b–f). Immunofluorescence results in the CA3 region further verified the results (Fig. 3g).

### Synaptic proteins decreased in hippocampi of Cx3cr1^CreER^IL-10^−/−^ mice

**Fig. 4:**
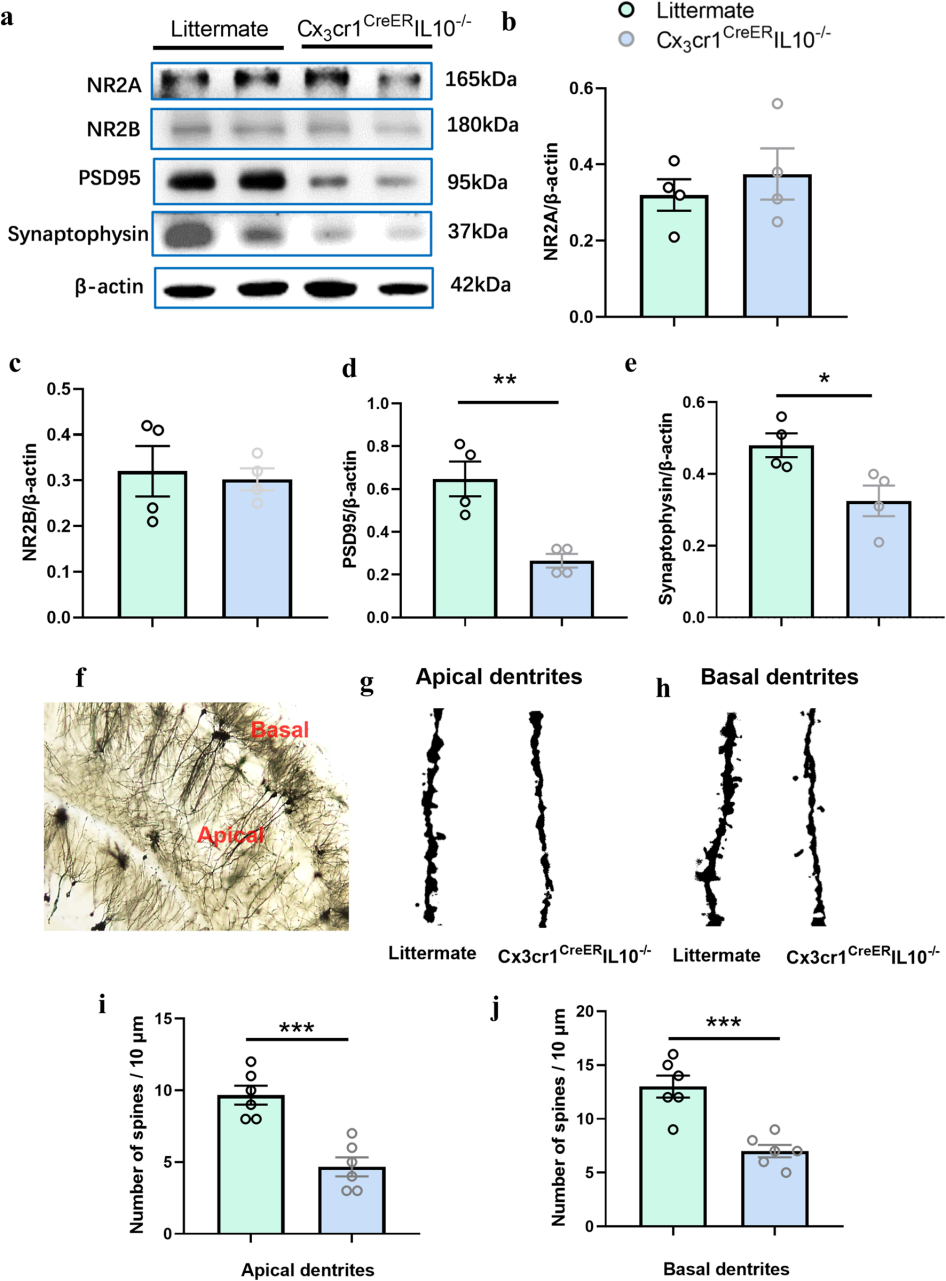
The expression of synaptic proteins in hippocampus of Cx3cr1^CreER^ IL-10^−/−^mice. **a** Western blotting bands of NR2A, NR2B, PSD95, and synaptophysin in littermates and in Cx3cr1^CreER^IL-10^−/−^mice. Bar graphs represent densitometric plots of protein expression in littermate and Cx3cr1^CreER^IL-10^−/−^mice in NR2A (**b**), NR2B (**c**), PSD95 (**d**), and synaptophysin (**e**). Each dot represents a mouse. Bars represent mean ± SEM. *n* = 4 in each group. Significant differences were established by *t*-test. Spine density of the littermates and the Cx3cr1^CreER^IL-10^−/−^ mice by Golgi staining (**f**). The spine density decreased in the pyramidal cells in both apical dendrites (**g** and **i**) and basal dendrites (**h** and **j**) in hippocampus. Scale (**l**). Images were captured with a 10 × objective (**f**) and 100 × objective (**g**). Bars represent mean ± SEM. *n* = 6 in each group. Significant differences were established by *t*-test. *P < 0.05, **P < 0.01, ***P < 0.001

Hippocampal synaptic proteins are one of the structural bases of spatial learning and memory. We tested the expression of glutamate receptors NR2A and NR2B, synaptic protein postsynaptic density protein 95 (PSD95), and synaptophysin in the hippocampus. In our results, NR2A and NR2B were not affected in the KO mice (*P* > 0.05, *n* = 4, *t* test, Fig. 4a–c), but PSD95 (*t* = 4.398, *P* = 0.005 < 0.01, *n* = 4, *t*-test) and synaptophysin (*t* = 2.850, *P* = 0.029 < 0.05, *n* = 4, *t*-test) were decreased in Cx3cr1^CreER^IL-10^−/−^ mice compared to their littermates, as determined by western blot analyses (Fig. 4a, d and e). The immunofluorescence results of PSD95 and synaptophysin in the CA3 region are shown in Additional file 1: Fig. S3, which was consistent with that of western blotting. The fluorescence intensity was reduced in both PSD95 and synaptophysin in the KO group. Golgi staining showed a significant decrease in the number of apical dendrites (*t* = 5.303, *P* = 0.0003 < 0.001, *n* = 6, *t*-test, Fig. 4g and i) and basal dendrites (*t* = 5.071, *P* = 0.0005 < 0.001, *n* = 6, *t*-test, Fig. 4h and j) in the hippocampus of the Cx3cr1^CreER^IL-10^−/−^ mice.

Synaptophysin is an integral membrane protein of small synaptic vesicles and has been identified as a useful marker for synaptic density [30]. PSD95 is a protein localized to the postsynaptic density of synapses [31] and plays a key role in synapse stabilization and plasticity [32], suggesting that microglia-derived IL-10 may play a role in synaptic protein synthesis through underlying mechanisms, thus affecting the spatial learning and memory ability of Cx3cr1^CreER^IL-10^−/−^ mice.

### Hippocampal inflammatory activity enhanced in Cx3cr1^CreER^IL-10^−/−^ mice

**Fig. 5:**
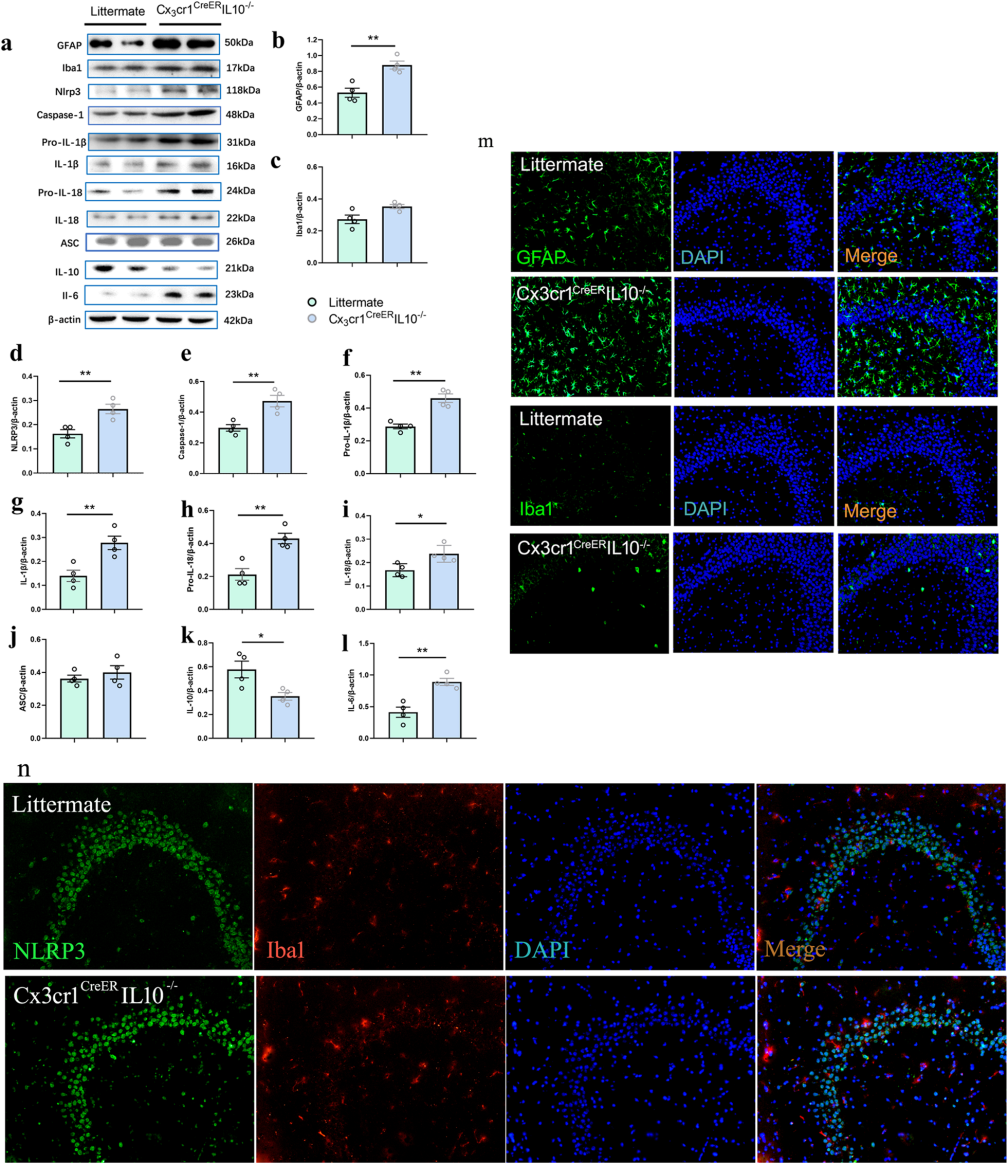
Neuroinflammatory activity enhanced in the hippocampus in Cx3cr1^CreER^IL-10^−/−^mice. **a** Western blotting bands of inflammation-related molecules in littermates and Cx3cr1^CreER^ IL-10^−/−^mice. Bar graphs represent densitometric plots of protein expression in littermate and Cx3cr1^CreER^IL-10^−/−^ mice in GFAP (**b**), Iba1 (**c**), NLRP3 (**d**), caspase-1 (**e**), Pro-IL-1β (**f**), IL-1β (**g**), Pro-IL-18 (**h**), IL-18 (**i**), ASC (**j**), IL-10 (**k**), and IL-6 (**l**). Each dot represents a mouse. Bars represent mean ± SEM. *n* = 4 in each group. Significant differences were established by *t*-test. **m** Expression of GFAP and Iba1 in CA3 region in mice hippocampal slices. Immunofluorescence images, captured with a 10 × objective. Green, immunoreactivity of GFAP and Iba1; blue, nuclei staining with DAPI. The merged images of GFAP, Iba1 and DAPI staining. **n** Immunofluorescence co-localization of NLRP3 and Iba1 in CA3 region in mice hippocampal slices. Immunofluorescence images, captured with a 10 × objective. Green, immunoreactivity of NLRP3; Red, immunoreactivity of Iba1; Blue, nuclei staining with DAPI. The merged images of NLRP3, Iba1 and DAPI staining. *P < 0.05, **P < 0.01

To determine whether changes in IL-10 level in microglia affect inflammation in vivo, the levels of glial marker Iba1 (for microglia) and GFAP (for astrocytes) were tested. Our results showed increased protein expression of GFAP (*t* = 4.583, *P* = 0.005 < 0.01, *n* = 4, *t*-test) and Iba1(*t* = 2.698, *P* = 0.036 < 0.05, *n* = 4, *t*-test, Fig. 5a–c), which was consistent with enhanced immunofluorescence levels of Iba1 and GFAP-positive cells in the hippocampal CA3 region (Fig. 5m). The increased expressions of GFAP and Iba1 suggested that the behavioral changes in KO mice were involved in inflammation activation.

We also investigated whether the inflammasome NLRP3 pathway was upregulated in the hippocampus. Protein expression of NLRP3 inflammasome components, NLRP3 (*t* = 3.976, *P* = 0.007 < 0.01, *n* = 4, *t*-test), caspase-1 (*t* = 4.055, *P* = 0.0067 < 0.01, *n* = 4, *t*-test), Pro-IL-1β (*t* = 5.784, *P* = 0.001 < 0.01, *n* = 4, *t*-test), IL-1β (*t* = 3.757, *P* = 0.009 < 0.01, *n* = 4, *t*-test), Pro-IL-18 (*t* = 4.562, *P* = 0.003 < 0.01, *n* = 4, *t*-test), IL-18 (*t* = 3.092, *P* = 0.02 < 0.05, *n* = 4, *t*-test) were significantly increased in the hippocampi of KO mice compared to their littermates (Fig. 5a, d–i, k and l). There was no change on the ASC protein expression (*t* = 0.820, *P* = 0.443 > 0.05, *n* = 4, *t*-test). In addition, our results showed that IL-10 (*t* = 2.922, *P* = 0.03 < 0.05, *n* = 4, *t*-test, Fig. 5k) decreased and IL-6 (*t* = 4.887, *P* = 0.003 < 0.01, *n* = 4, *t*-test, Fig. 5l) increased in the hippocampi of Cx3cr1^CreER^IL-10^−/−^ mice. Immunofluorescence co-localization of NLRP3 and Iba1 showed that the increase of NLRP3 was not mainly located in microglia (Fig. 5n).

### TRPC5 down-regulated in excitatory neurons in the hippocampi of Cx3cr1^CreER^IL-10^−/−^ mice

**Fig. 6:**
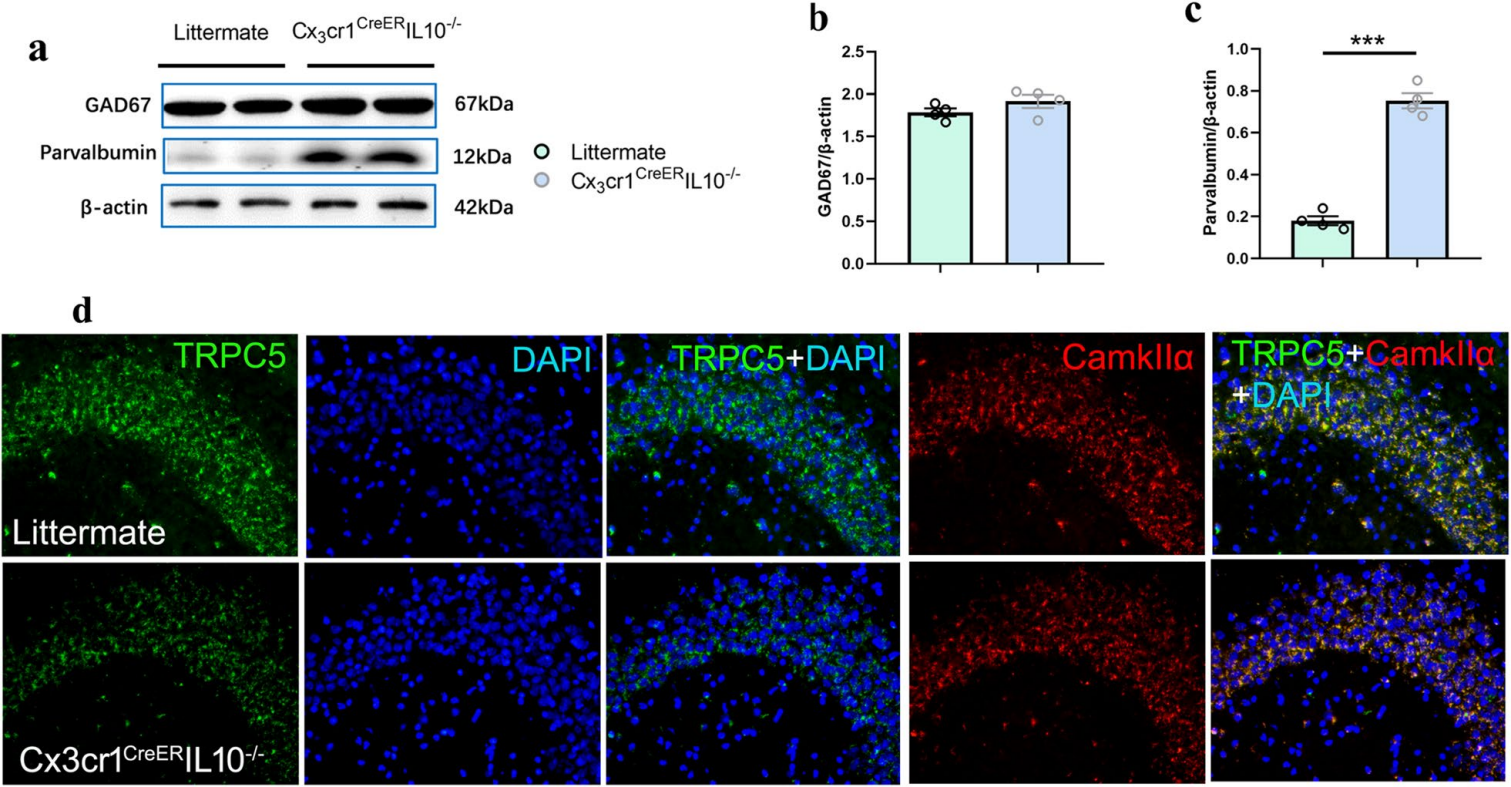
The effects of knocking out IL-10 from Microglia in interneuron and the increase of TRPC5 in excitatory neuron. **a** Western blotting bands of GAD67 and parvalbumin in littermates and in Cx3cr1^CreER^ IL-10^−/−^mice. Bar graphs represent densitometric plots of protein expression in littermate and Cx3cr1^CreER^IL-10^−/−^mice in GAD67 (**b**) and parvalbumin (**c**). Each dot represents a mouse. Bars represent mean ± SEM. *n* = 4 in each group. Significant differences were established by *t*-test. **d** Colocalization of TRPC5 and CaMKIIα in CA3 region in hippocampal slices. Immunofluorescence images, captured with a 20 × objective. Green, immunoreactivity of TRPC5; red, immunoreactivity of CaMKIIα, a marker of excitatory neurons, blue, nuclei staining with DAPI. The merged images of TRPC5, CaMKIIα and DAPI staining. ***P < 0.001

The balance between the central inhibitory and facilitatory systems may serve as a principal mechanism in memory activation and regulation [33]. TRPC is expressed in both the inhibitory and excitatory neurons of the CNS [34]. We explored how downregulation of TRPC in Cx3cr1^CreER^IL-10^−/−^ mice affects memory by affecting network excitation and inhibitory balance. Our results showed that gene KO did not affect the content of GAD67 (*t* = 1.430, *P* = 0.20 > 0.05, *n* = 4, *t*-test, Fig. 6a, b), but could enhance the content of Pv (*t* = 13.53, *P* < 0.0001, *n* = 4, *t*-test, Fig. 6a, c), which is a specific subtype of gamma aminobutyric acid (GABA) interneurons that may subserve distinct behavioral functions and behavior-dependent network activities [35]. Inhibition of the GABAergic system has memory-facilitating effects, whereas stimulation produces memory impairment. These results suggest that the effect of TRPC4 or 5 downregulation on learning and memory in Cx3cr1^CreER^IL-10^−/−^ mice mainly acted on excitatory neurons. Immunofluorescence showed increased TRPC5 co-localization in the hippocampus with CaMKIIα (an excitatory neuron marker) (Fig. 6d).

### Upregulation of TRPC5 in excitatory neurons can improve memory impairment in Cx3cr1^CreER^IL-10^−/−^ mice

**Fig. 7:**
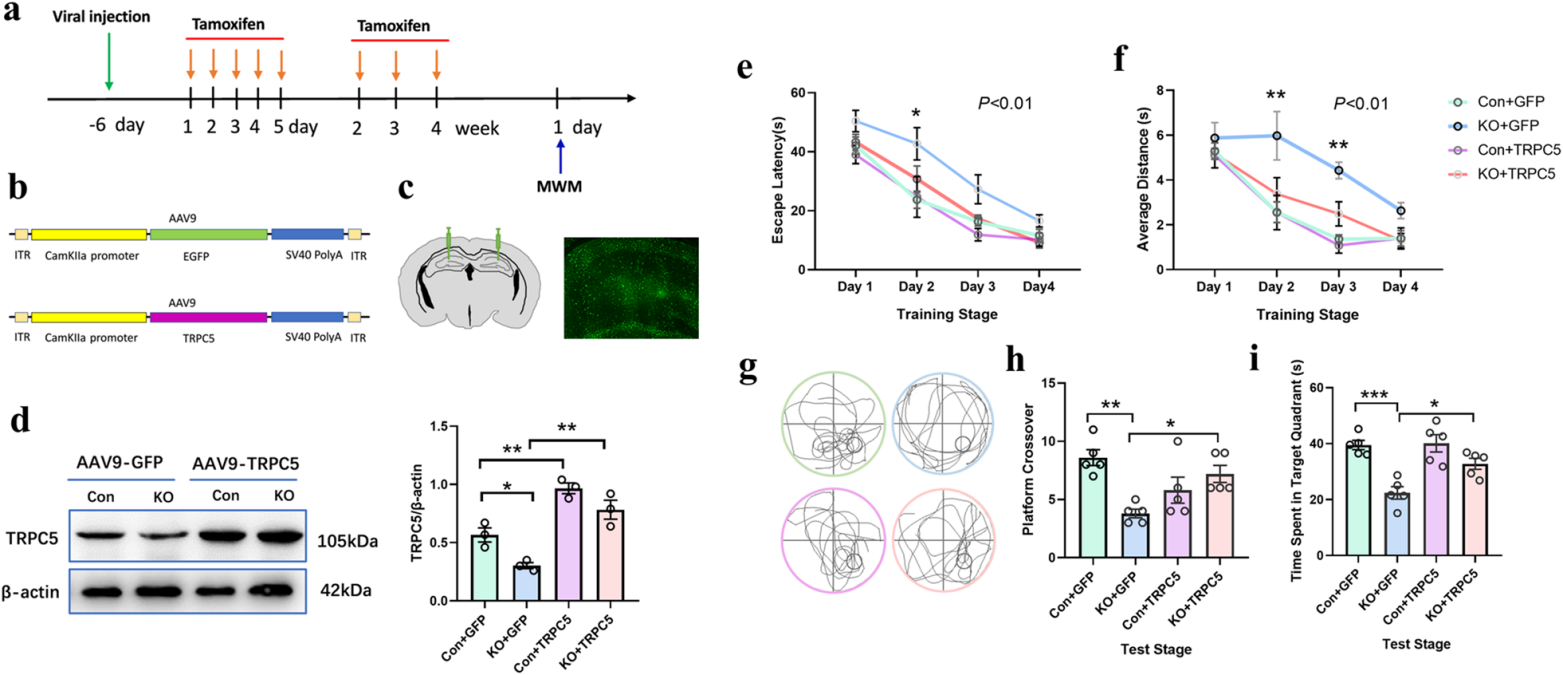
Upregulation of TRPC5 in hippocampal excitatory neurons improving the learning and memory ability of Cx3cr1^CreER^ IL-10^−/−^mice. **a** Schematic of experimental design and schedule. The animal experimental protocol indicated the time course of various interventions utilized during the experiment. **b**, **c** Schematics of AAV9-CaMKIIα-TRPC5 (**b**) and stereotaxic injection in the hippocampus (**c**). **d** The expression level of TRPC5 in hippocampus by Western blotting after AAV9 injection (*n* = 3). There was a significant increase in both escape latency (**e**) and average distance (**f**) in KO + TRPC5 compared to the KO group in the training stage. **g** Representative movement traces from the four groups on the test stage of the MWM task. The mice in the KO + TRPC5 group had more convergent paths in the test stage in the target quadrant, suggesting memory improvements. There was a significant increase in both the platform crossover times (**h**) and time spent in the target quadrant (**i**) in the KO + TRPC5 group in the test stage compared with the KO group. Each dot represents a mouse. Bars represent mean ± SEM. *n* = 5 in each group, Significant differences were established by two-way ANOVA (**e**, **f**) and one-way ANOVA (**h**, **i**). *P < 0.05, **P < 0.05

CaMKIIα was used as the promoter of the AAV9 virus, specifically upregulating TRPC5 in excitatory neurons (Fig. 7b, c). The expression of TRPC5 after AAV9 injection in hippocampus were tested by western blotting [*F* (3, 8) = 24.99, *P* < 0.001, Fig. 7d]. After injection of the virus for 4 weeks, we performed MWM tests in all groups of mice. The results of MWM showed differences in escape latency to the platform [*F* (3, 16) = 8.137, *P* < 0.01] and average swimming distance [*F* (3, 16) = 13.00, *P* < 0.001] during the learning phase. The KO mice treated with AAV-9 TRPC5 injection showed an improved behavioral performance compared to KO-GFP group (*P* < 0.05, Fig. 7e, f). In the training stage, the KO mice treated with TRPC5 exhibited alleviated memory damage in both platform crossover times [*F* (3, 16) = 7.053, *P* < 0.01, Fig. 7h] and in time spent in the target quadrant [*F* (3, 16) = 12.81, *P* < 0.001, Fig. 7i].

### The effects of TRPC5 on Cx3cr1^CreER^IL10^−/−^ mice by suppressing NLRP3-associated neuroinflammation

**Fig. 8:**
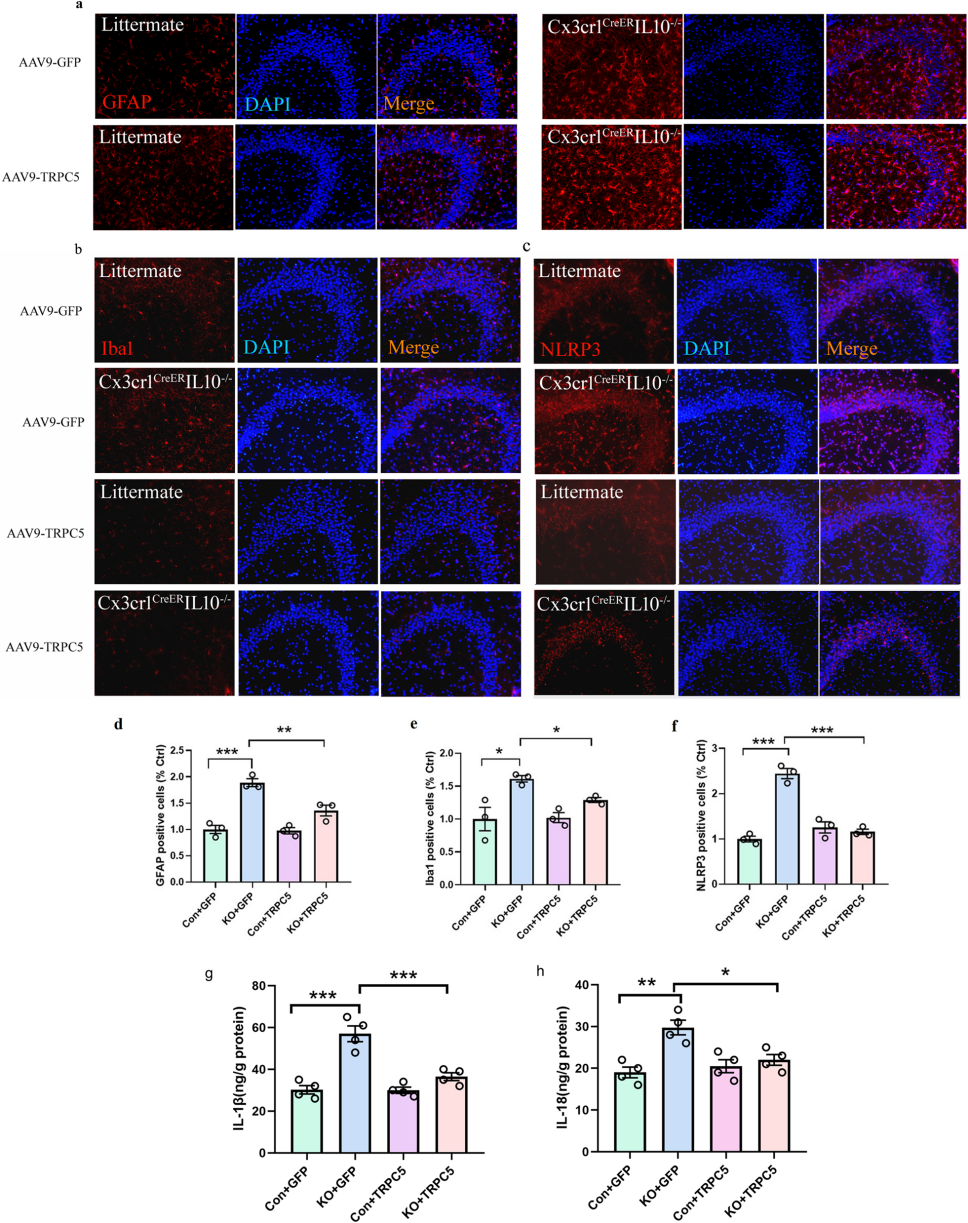
Upregulation of TRPC5 in hippocampal excitatory neurons inhibiting the neuroinflammation in hippocampus. **a**–**c** Expression of GFAP (**a**), Iba1 (**b**), and NLRP3 (**c**) in the CA3 region of hippocampal slices. Immunofluorescence images were captured with a 10 × objective. Red, immunoreactivity of GFAP, Iba1, and NLRP3; blue, nuclei stained with DAPI. The merged images of GFAP, Iba1 and DAPI staining. Bars represent positive cells in GFAP (**d**), Iba1 (**e**) and NLRP3 (**f**) at mean ± SEM. *n* = 3 in each group. Bars showed the levels of IL-1β (**g**) and IL-18 (**h**) by ELISA test in the hippocampus at mean ± SEM. *n* = 4 in each group. Significant differences were established by one-way ANOVA. *P < 0.05, **P < 0.01, ***P < 0.001

The mechanism of TRPC5 upregulation in excitatory neurons contributes to the improvement behavioral performance in KO mice. We used immunofluorescence to detect GFAP, Iba1, and NLRP3 in the brain regions of the hippocampus. The results showed that increasing the expression of TRPC5 inhibited the number of GFAP [*F* (3,8) = 28.01, *P* < 0.001, Fig. 8a, d], Iba1 [*F* (3,8) = 8.011, *P* < 0.01, Fig. 8b, e] and NLRP3 [*F* (3,8) = 49.59, *P* < 0.001, Fig. 8c, f] positive cells in the hippocampus, especially in the CA3 region. We then tested NLRP3-related cytokines IL-1β and IL-18 in the hippocampus by ELISA, and the results showed that increased TRPC5 expression in KO group down-regulated both the expression levels of IL-1β [*F* (3,12) = 2.773, *P* < 0.001, Fig. 8g] and IL-18 [*F* (3,12) = 0.360, *P* < 0.01, Fig. 8h].

## Discussion

IL-10 is a key cytokine that represses excessive inflammatory responses and is linked to anti-inflammatory and protective functions in the CNS [36]. In the CNS, IL-10 is mainly produced by astrocytes and microglia. Our results showed that Cx3cr1^CreER^IL-10^−/−^ mice showed impaired spatial cognition, suggesting that microglia-targeted production of IL-10 plays an important role in hippocampal function. This result is similar to the results of other relevant studies, in which IL-10^tm1/tm1^ male mice with a low expression of IL-10 exhibited defective learning and memory behaviors in the MWM test [7]. A recent study showed that IL-10 produced from microglial cells in non-learned helpless mice is necessary to maintain learning and memory [13]. Intranasal administration of IL-10 increased dendritic spine density by 2.0- and 4.3-fold in the dentate gyrus of non-learned helpless and learned helpless mice [13].

We detected mRNA levels of TRPC subtypes in the hippocampus other than TRPC2, a pseudogene in humans. TRPC1, 3, 4, and 5 mRNA levels decreased. Further results confirmed that TRPC4 and TRPC5 protein levels were down-regulated. These results can be linked to our recent study, in which the protein expression of TRPC5 is down-regulated in the medial prefrontal cortex and amygdala in Cx3cr1^CreER^IL-10^−/−^ mice [27]. In other researches, recombinant IL-10 results in the decreased expression of N-cadherin, which interacts with TRPC4 and TRPC6 during the formation of stress fiber [37]. IL-10 produced in B lymphocytes depends on the upregulation of TRPC1 protein [38]. These studies provide clues to the interaction between IL-10 and TRPC channels.

The KO mouse showed a poor performance in MWM with the decrease of TRPC4 and TRPC5 in hippocampus. This result could be linked to another related study, in which the TRPC1/4/5 channels were relevant to synaptic transmission for working memory formation and in relearning tasks in the hippocampus [3]. Controversially, TRPC1/4/5 KO did not affect mouse reference memory in that study. Based on the synaptic plasticity deficit, it could be difficult to explain how TRPC1/4/5 KO can specifically affect working memory without affecting the reference memory [3]. Notably, during the training phase in the MWM task, TRPC1/4/5^−/−^ mice showed a similar decrease in learning ability. This difference may also be related to the interaction of TRPC subtypes, since the expression of TRPC1 protein in our study did not change in mice.

The cognitive impairment caused by Cx3cr1^CreER^IL-10^−/−^ mice is closely related to a decrease in synaptic transmission. Synaptic transmission involves the release of neurotransmitters from presynaptic neurons, which then bind to specific postsynaptic receptors. Synaptic proteins PSD95 and synaptophysin were tested in our study, and an obvious decrease in presynaptic synaptophysin and postsynaptic density protein PSD95 suggested that the memory deficits of Cx3cr1^CreER^IL-10^−/−^ mice depend on structural changes in synaptic associated proteins, which is a dual mechanism involving presynaptic and postsynaptic processes. Notably, the glutamate receptors NR2A and NR2B were not involved in this process.

IL-10 is a key cytokine that has been shown to inhibit excessive inflammation associated with anti-inflammatory and protective functions in the CNS. IL-10 exerts anti-inflammatory effects by inhibiting monocyte/macrophage-derived cytokines, including TNF-*α*, IL-1β, IL-6, and IL-18. It has been reported that immune dysfunction is commonly associated with the progression of many CNS diseases, such as neuropsychiatric disorders and neurodegenerative disorders [36, 39]. Moreover, the role of cytokines is of particular interest because they are involved in cognitive impairment in hippocampal-dependent memory [40].

The increase in the number of GFAP- and Iba1-positive cells indicates an imbalance in local neuroinflammation in the hippocampus. Furthermore, Cx3cr1^CreER^IL-10^−/−^ mice exhibited a marked upregulation of NLRP3 in hippocampus, and accordingly caspase-1 increase and subsequent IL-1β and IL-18 processing and release, compared with littermate mice, accompanied by the downregulation of IL-10 and upregulation of IL-6. These results suggest that KO IL-10 from microglia may affect the behavioral performance of animals by altering the balance of local pro-inflammatory and anti-inflammatory networks. The NLRP inflammasome has been identified as a multi-protein complex that plays a pathogenic role in nervous system diseases. Among these types of inflammasomes, NLRP3 has been implicated in several chronic inflammatory responses and is associated with many CNS diseases [41]. Baicalin increases the performance of APP/PS1 transgenic mice in MWM by suppressing NLRP3 inflammasomes to alleviate microglia-mediated neuroinflammation [42]. Activation of the NLRP3 inflammasome plays a role in the gastrodin-induced amelioration of cognitive impairment in diabetic rats [43], and NLRP3 inflammation, a molecular marker involved in inflammatory response, is known to play a key role in the development of cognitive impairment.

TRPC channels are widely expressed in the brain and are related to a variety of neuronal functions [44], but in general TRPC4 and TRPC5 are the predominant subtypes in the rodent brain [45]. In our study, we observed that both TRPC4 and 5 decreased in hippocampi of KO mice, which indicated that both channels could contribute to the spatial memory impairment of Cx3cr1^CreER^IL-10^−/−^ mice, although it is difficult to judge which of these channels contributes to cognitive impairment. TRPC5 is highly expressed in the hippocampus [46, 47]. TRPC channels have been implicated in presynaptic and postsynaptic neuronal processes. To date, the physiological function of TRPC channels in the brain is unknown. In cultured neurons, TRPC5 insertion and TRPC5-mediated Ca^2+^ influx are important determinants of hippocampal neurite growth rate and growth cone morphology [48, 49].

In our study, we used CaMKIIα as a promoter to specifically express TRPC5 in pyramidal neurons. The results showed that high expression of TRPC5 could improve spatial cognitive impairment in IL-10 KO mice. Furthermore, the results suggested that the effect of TRPC5 on behavioral improvement in IL-10 KO mice might be related to its inhibition of neuroinflammation. Increasing the expression of TRPC5 in excitatory neurons can reduce the high expression of GFAP and Iba1 in Cx3cr1^CreER^IL-10^−/−^ mice. Several other studies have also focused on the role of TRPC5 in inflammation. Growing evidence has linked the activation of TRPC5 complexes to inflammation. TRPC5^−/−^ mice showed enhanced synovitis and local inflammation, and the TRPC4/5 antagonist ML204 increased the levels of TNF-α and IL-10 in synovial fluid. In TRPC5 KO and wild-type mice treated with TRPC4/5 antagonists, IL-10 secretion was found to be elevated to regulate a highly inflammatory response. The absence or antagonism of TRPC5 increases the local secretion of many key pro-inflammatory cytokines, such as TNF-α and IL-1β [50]. TRPC5^−/−^ mice pretreated with thioredoxin also showed that cytokines, TNF-α and IL-6, in the peritoneum were exacerbated in the systemic inflammatory response [51]. There have also been conflicting studies on the relationship between TRPC5 and inflammation. Expression of TRPC5 in nasal polyps was positively correlated with the number of eosinophils, IL-6 expression and inflammation [52], suggesting that these pathways may respond differently to different inflammatory responses. Immunofluorescence co-localization of NLRP3 and Iba1 showed the increase of NLRP3 without co-localization with microglia in KO mice. The results suggested that the increase of NLRP3 could depend mainly on excitatory neurons, which make up the majority of the cell population. According to Py et al.’s study [53], TRPC1 was identified as a substrate for caspase-11, which regulates inflammatory responses, and TRPC1 deficiency could increase IL-1 β secretion which depended on the NLRP3 activator triggering. These results provide some clues for the downregulation of NLRP3 by TRPC5-AAV9 injection in excitatory neurons. However, the specific mechanism needs to be further studied.

TRPC5 may also directly affect the learning and memory ability of animals by improving the efficiency of synaptic transmission. Some studies have shown that TRPC5 regulates synaptic plasticity by changing the presynaptic Ca^2+^ homeostasis of hippocampal neurons [54] and both TRPC4 and 5 channels contribute to persistent firing in CA1 pyramidal cells [55]. In another study, TRPC5 channels, profoundly regulate synaptic plasticity and elevate the rate of spontaneous release, indicating a key role of TRPC5 in short-term plasticity. In addition, the specific activation of TRPC4/5 induced a significant increase in the mEPSC frequency in hippocampal neurons of wild-type mice [54]. TRPC5 is also an important determinant at neurite outgrowth rates, growth cone morphology [49] and plateau potentials of excitatory neurons in the hippocampus [56].

## Conclusions

Our results using Cx3cr1^CreER^IL-10^−/−^mice indicated the involvement of TRPC4 and 5 channels in recognition impairment. Specific high expression of the excitatory neuron TRPC5 can improve the behavioral performance of KO mice. However, a limitation in our present study does not preclude the role of TRPC4 in the process. Future research should focus on resolving the observations mentioned above and determining the molecular mechanisms between TRPC and the inflammasome NLRP3 system. In addition, it is also essential to clarify how TRPC interacts with NLRP3 affect the behavioral performance in the KO mouse. In a specific environment, an attempt should be made to clarify the relationship between the cooperative compensation of various subtypes of TRPC functions and the behavioral representation of animals.

## Supplementary Information

**Additional file 1.** **Fig. S1** Identification of knockout mice by PCR analysis. PCR analysis in Cx3cr1wt/wt and Cx3cr1wt/CreER mice. **a**. PCR analysis for Flox homozygous. **b**. PCR analysis for Cre heterozygous. **c**. PCR analysis for FACS-purified macrophages in blood 7 d after final tamoxifen treatment for the presence of conditional undeleted (1165 bp) or deleted IL-10 alleles (558 bp). **d**. A schematic of the DNA assembly, location of Cre, IL-10 gene and other components, along with nucleotide size before and after disruption. **e**. Immunofluorescence images, captured with a 10× objective, Green, Iba1; Red, immunoreactivity of IL-10; blue, nuclei staining with DAPI. Merged images of Iba1, IL-10 and DAPI stain. The arrow marks showed the co-location of the Iba1 and IL-10. **f**. Western bolting analysis of FACS-purified littermates and Cx3cr1CreERIL10-/- cerebral cortex microglia 7 d after tamoxifen. **Fig. S2**. Cx3cr1CreER IL-10-/-mice shown a decrease recognition impairment in RMWM task. **a**: Representative movement traces from two groups on the training stage of RMWM task. Cx3cr1CreERIL-10-/-mice had more dispersed paths in training stage, suggesting learning ability impairments. There was a significant increase both in escape latency (**b**) and in average distance (**c**) in Cx3cr1CreERIL-10-/- group in training stage, while both groups of mice swim at the same speed (**d**). **e**: Representative movement traces from two groups on the test stage of RMWM task. There was no significant difference in platform crossover times (**f**), time in target quadrant (**g**) and swimming speed (**h**). Each dot represents a mouse. Bars represent mean±SEM. n = 8 in each group. Significant differences were established by two-way ANOVA (**b**-**d**) and t-test in other bar graphs, *P<0.05. **Fig. S3**. Immunofluorescence staining results of synaptic proteins in hippocampal CA3 region. **a**: Expression of PSD95 and synaptophysin in the CA3 region of mouse hippocampal slices. Immunofluorescence images were captured with a 20× objective, green, immunoreactivity of PSD95 and synaptophysin; blue, nuclei stained with DAPI. The merged images of PSD95, synaptophysin, and DAPI staining. There was a decrease in the expression of PSD95 and synaptophysin in the hippocampus of Cx3cr1CreERIL-10-/-mice. **b**: Immunofluorescence images, captured with a 20× objective. Green, immunoreactivity of NR2A and NR2B; blue, nuclei staining with DAPI. Merged images of NR2A, NR2B and DAPI stain. There was no difference in the expression of NR2A and NR2B in the hippocampus of two groups.

## Data Availability

The datasets used during the current study are available from the corresponding author on reasonable request.

## Abbreviations

TRPC: Transient receptor potential canonical
IL-10: Interleukin-10
GFAP: Glial fibrillary acidic protein
Iba1: Ionized calcium-binding adaptor molecule 1
PSD95: Postsynaptic density protein 95
NLRP3: Nod-like receptor protein 3
CaMKII: Calmodulin-dependent protein kinase II
LTP: Long-term potentiation
CNS: Central nervous system
MWM: Morris water maze
NOR: Novel object recognition
Olr: Object location recognition
DI: Discrimination index
PVDF: Polyvinylidene difluoride
Pv: Parvalbumin
DG: Dentate gyrus
ANOVA: Analysis of variance
NLRP: Nod-like receptor pyrin domain containing
AD: Alzheimer’s disease
KO: Knock out
mEPSC: Miniature excitatory postsynaptic current
