# Supplementary material for: Upregulation of TRPC5 in hippocampal excitatory synapses improves memory impairment associated with neuroinflammation in microglia knockout IL-10 mice
Source: J Neuroinflammation. 2021 Nov 26;18:275. doi: 10.1186/s12974-021-02321-w (PMC8620645; doi:10.1186/s12974-021-02321-w)
Supplement: Supplementary file 1 — Additional file 1. Fig. S1 Identification of knockout mice by PCR analysis. PCR analysis in Cx3cr1wt/wt and Cx3cr1wt/CreER mice. a. PCR analysis for Flox homozygous. b. PCR analysis for Cre heterozygous. c. PCR analysis for FACS-purified macrophages in blood 7 d after final tamoxifen treatment for the presence of conditional undeleted (1165 bp) or deleted IL-10 alleles (558 bp). d. A schematic of the DNA assembly, location of Cre, IL-10 gene and other components, along with nucleotide size before and after disruption. e. Immunofluorescence images, captured with a 10× objective, Green, Iba1; Red, immunoreactivity of IL-10; blue, nuclei staining with DAPI. Merged images of Iba1, IL-10 and DAPI stain. The arrow marks showed the co-location of the Iba1 and IL-10. f. Western bolting analysis of FACS-purified littermates and Cx3cr1CreERIL10-/- cerebral cortex microglia 7 d after tamoxifen. Fig. S2. Cx3cr1CreER IL-10-/-mice shown a decrease recognition impairment in RMWM task. a: Representative movement traces from two groups on the training stage of RMWM task. Cx3cr1CreERIL-10-/-mice had more dispersed paths in training stage, suggesting learning ability impairments. There was a significant increase both in escape latency (b) and in average distance (c) in Cx3cr1CreERIL-10-/- group in training stage, while both groups of mice swim at the same speed (d). e: Representative movement traces from two groups on the test stage of RMWM task. There was no significant difference in platform crossover times (f), time in target quadrant (g) and swimming speed (h). Each dot represents a mouse. Bars represent mean±SEM. n = 8 in each group. Significant differences were established by two-way ANOVA (b-d) and t-test in other bar graphs, *P<0.05. Fig. S3. Immunofluorescence staining results of synaptic proteins in hippocampal CA3 region. a: Expression of PSD95 and synaptophysin in the CA3 region of mouse hippocampal slices. Immunofluorescence images were captured with a 20× objective, gree [file 12974_2021_2321_MOESM1_ESM.docx]

**Fig.S1**


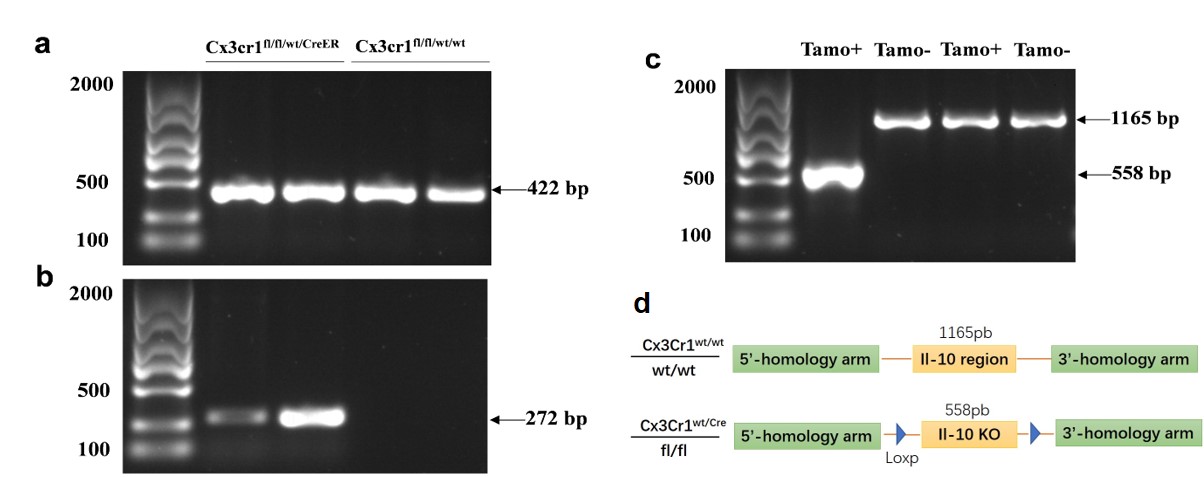


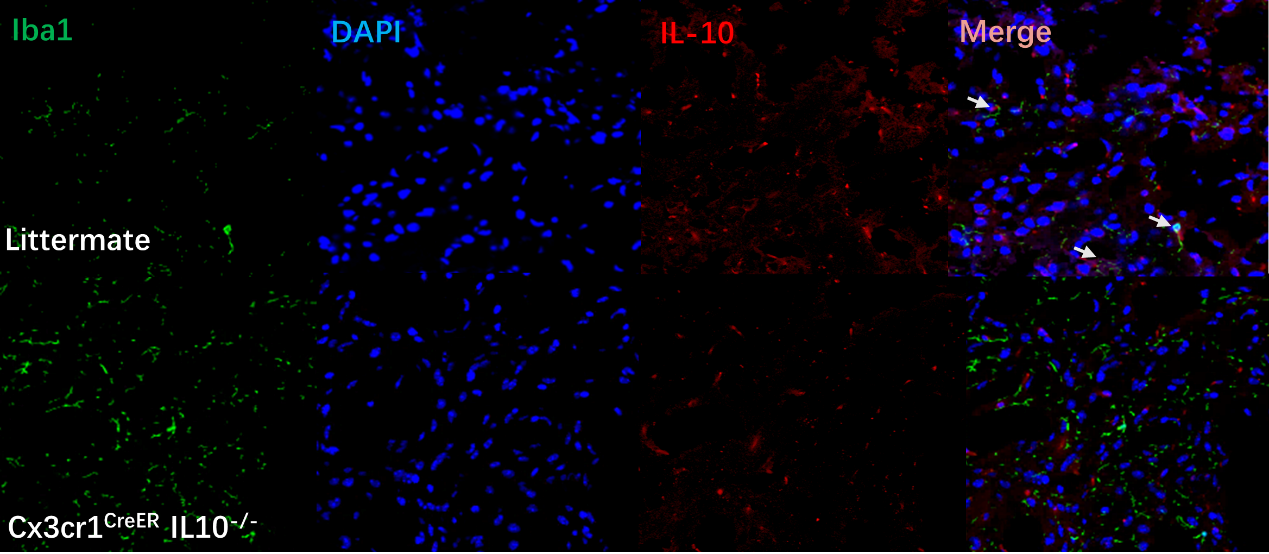
  **e**

**
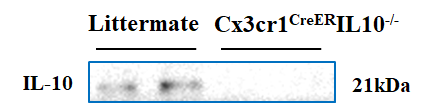
 f**

**Fig.S1 Identification of knockout mice by PCR analysis.**

PCR analysis in Cx3cr1^wt/wt^ and Cx3cr1^wt/CreER^ mice. a. PCR analysis for Flox homozygous. b. PCR analysis for Cre heterozygous. c. PCR analysis for FACS-purified macrophages in blood 7 d after final tamoxifen treatment for the presence of conditional undeleted (1165 bp) or deleted IL-10 alleles (558 bp).d.A schematic of the DNA assembly, location of Cre, IL-10 gene and other components, along with nucleotide size before and after disruption. e. Immunofluorescence images, captured with a 10× objective, Green, Iba1; Red, immunoreactivity of IL-10; blue, nuclei staining with DAPI. Merged images of Iba1, IL-10 and DAPI stain. The arrow marks showed the co-location of the Iba1 and IL-10. f.  Western bolting analysis of FACS-purified littermates and Cx3cr1^CreER^IL10^-/-^ cerebral cortex microglia 7 d after tamoxifen.

**Fig.S2**


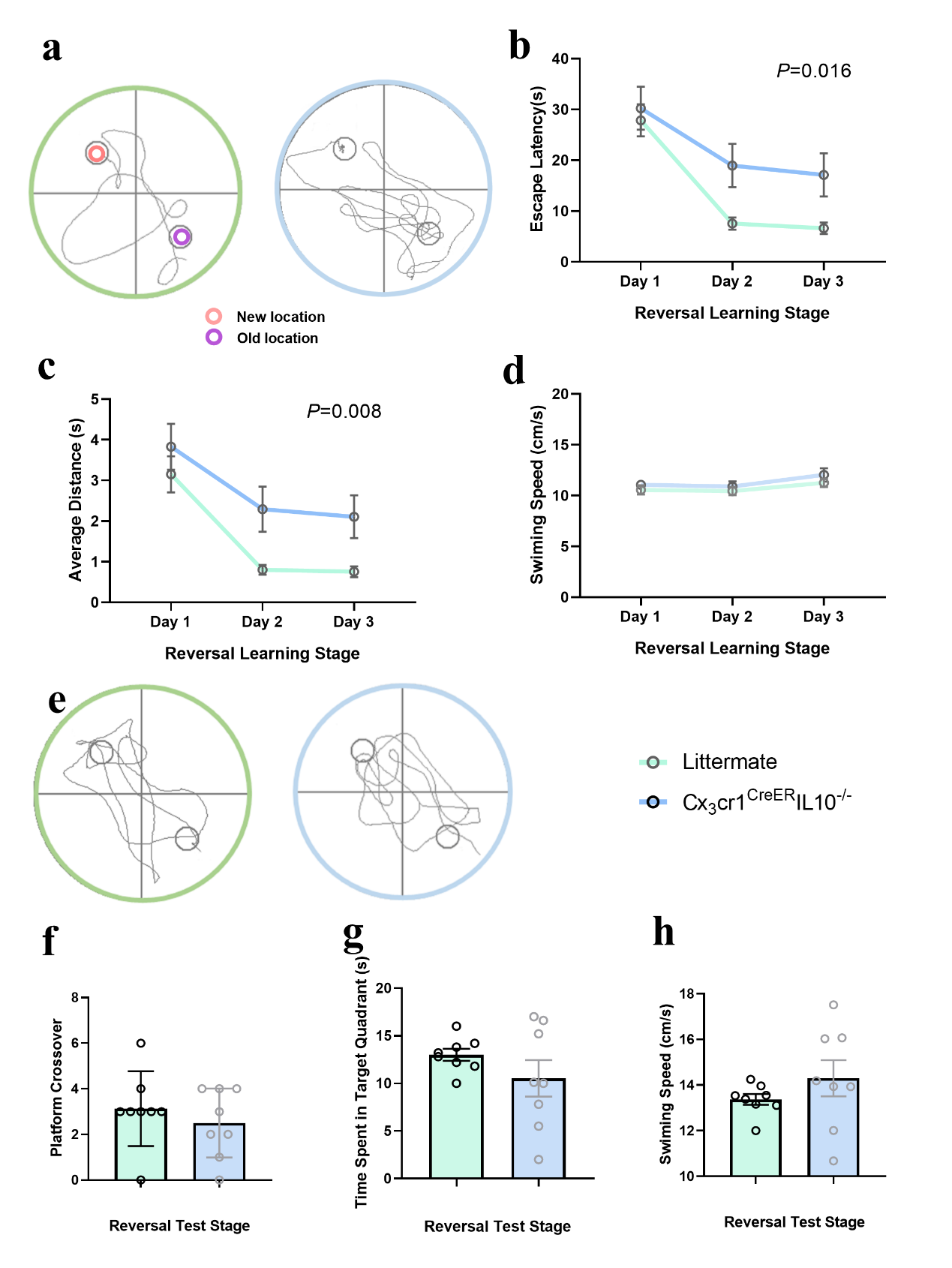


**Fig.S2. Cx3cr1^CreER^ IL-10^-/-^mice shown a decrease recognition impairment in RMWM task.**

**a: Representative movement traces from two groups on the training stage of RMWM task.** Cx3cr1^CreER^IL-10^-/-^**mice had** more dispersed paths in training stage, suggesting learning ability impairments**.** There was a significant increase both in escape latency (b) and in average distance(c) in Cx3cr1^CreER^IL-10^-/-^ **group in training stage, while both groups of mice swim at the same speed(d). e: Representative movement traces from two groups on the test stage of RMWM task. There was no significant difference in platform crossover times (f), time in target quadrant (g) and swimming speed (h).** Each dot represents a mouse. Bars represent mean±SEM. *n*=8 in each group. Significant differences were established by two-way ANOVA (b-d) and *t-test* in other bar graphs, **P*<0.05.

**Fig.S3**

**
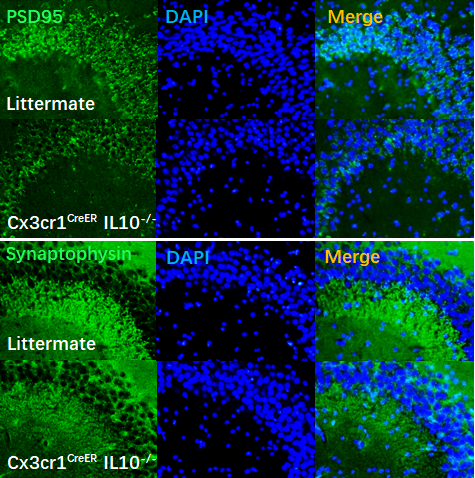
**


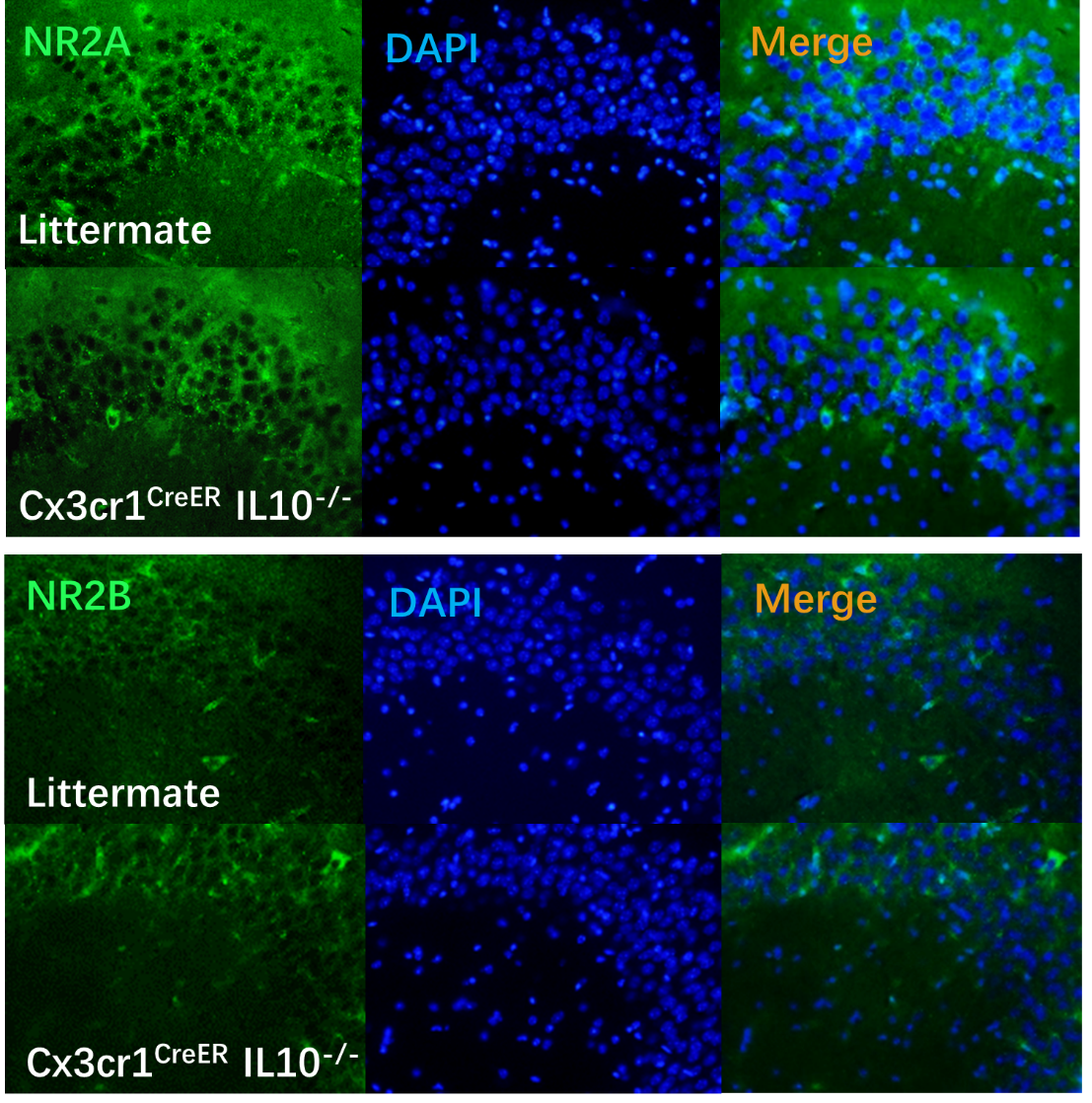


**Fig.S3. Immunofluorescence staining results of synaptic proteins in hippocampal CA3 region.**

a: Expression of PSD95 and synaptophysin in the CA3 region of mouse hippocampal slices. Immunofluorescence images were captured with a 20× objective, green, immunoreactivity of PSD95 and synaptophysin; blue, nuclei stained with DAPI. The merged images of PSD95, synaptophysin, and DAPI staining. There was a decrease in the expression of PSD95 and synaptophysin in the hippocampus of Cx3cr1^CreER^IL-10^-/-^**mice**. b: Immunofluorescence images, captured with a 20× objective. Green, immunoreactivity of NR2A and NR2B; blue, nuclei staining with DAPI. Merged images of NR2A, NR2B and DAPI stain. There was no difference in the expression of NR2A and NR2B in the hippocampus of two groups.
